# Nanocomposites for Machining Tools

**DOI:** 10.3390/ma10101171

**Published:** 2017-10-13

**Authors:** Daria Sidorenko, Pavel Loginov, Leon Mishnaevsky, Evgeny Levashov

**Affiliations:** 1 Scientific-Educational Center of SHS, National University of Science and Technology “MISIS”, Leninsky prospekt 4, 119049 Moscow, Russia; pavel.loginov.misis@list.ru (P.L.); levashov@shs.misis.ru (E.L.); 2 Department of Wind Energy, Technical University of Denmark, Frederiksborgvej 399, 4000 Roskilde, Denmark; lemi@dtu.dk

**Keywords:** machining, cemented carbide, steel, ceramics, superhard materials, diamond, nanocomposite, micromechanical modelling

## Abstract

Machining tools are used in many areas of production. To a considerable extent, the performance characteristics of the tools determine the quality and cost of obtained products. The main materials used for producing machining tools are steel, cemented carbides, ceramics and superhard materials. A promising way to improve the performance characteristics of these materials is to design new nanocomposites based on them. The application of micromechanical modeling during the elaboration of composite materials for machining tools can reduce the financial and time costs for development of new tools, with enhanced performance. This article reviews the main groups of nanocomposites for machining tools and their performance.

## 1. Introduction

Machining tools are the most important means of production. They need to be characterized by high productivity, wear-resistance and technological effectiveness. The quality of the tools plays an important role in the machine-building and energy sectors, and many other industries [1]. The machining industry is an integral part of the production sector, and its development directly affects the state of the economy of countries and regions. The diversity of manufactured tools permits cutting, drilling, whetting, milling, profiling, honing and superfinishing of various materials with high productivity.

The world consumption of machining tools increased dramatically from 2003 to 2011, and today remains approximately at the same level: at present, the annual consumption of the tools exceeds $50 billion [2,3]. High-speed steels, cemented carbides, ceramics and metalloceramics as well as superhard materials (SHMs), such as diamond, cubic boron nitride (CBN) or composite materials based on them, are the most widespread materials used in machining tools. The intense development of machinery requires the development of new tool materials with enhanced performance qualities. A combination of increased hardness, toughness and wear-resistance of tool materials [4] is the main goal when it comes to devising tools with enhanced service characteristics. There is a new trend that consists in the development of nanostructured alloys and composite materials with nanosized structural constituents.

Nanocomposites with a metallic matrix have a substantial advantage over traditional materials by virtue of higher values of elastic modulus, strength, wear-resistance, and thermal stability [5,6,7]. Besides enhancing their mechanical qualities, nanomodification permits improvements in the thermal conductivity and corrosion resistance [8] of the metallic matrices [9].

The phase composition, size and shape of the particles [10], their concentration, uniformity of distribution within the volume of the matrix, and the nature of interaction of the nanomodifiers with the matrix, all affect the properties of the composite. Depending on the purpose of the material, hard (SiC, WC, Al_2_O_3_, TiC, etc.) or soft (hexagonal boron nitride (hBN), MoS_2_, graphite, etc.) particles can be used for the reinforcing phase. The reinforcing nanoparticles can be divided into continuous (fibers) and discontinuous ones (whiskers and particles).

Reinforcement with dispersed particles is applied not only in three-dimensional materials, but also in coatings. The nanomodification considerably increases the coatings hardness as well as abrasion- and corrosion-resistance [11,12].

Development of the nanoindustry and the growing requirements of industry to ensure the high performance of composite materials has led to appearance of a new class of hybrid metal matrix composites, obtained by adding two or more nanomodifying components of different composition and shape with different properties to an initial matrix [13,14], with each component performing a certain function. For example, a graphite component (nanofibers, nanotubes, graphene) enhances their tribological characteristics [15,16]; and a hard and non-deforming component (tungsten carbide, zirconium oxide, titanium carbide, etc.) ensures considerable enhancement of their mechanical properties. In [17,18,19], authors successfully applied hybrid modification with micro- and nanosized particles. When alloyed with dispersed particles, the enhancement of mechanical properties of a metal matrix composite (MMC) is explained by three mechanisms: the Hall–Petch effect (by means of decreasing grain size) [20]; the Orowan effect [21]; and reinforcement related to the difference between the thermal expansion coefficients of the matrix materials and reinforcing particles [22].

## 2. Nanocomposites in Machining Tools

Development of nanocomposites for machining tools is a constantly growing scientific and industrial area. Nanocomposites are successfully applied in each of the five large classes of materials for machining tools: high-speed steels, ceramics, cemented carbides, superhard materials, and coatings. For the first time, such an approach was applied during the development of high-speed tool steels [23]. Alloying common carbon steel with tungsten, molybdenum, chromium, vanadium and other carbide-forming elements, as well as quenching and tempering at special regimes, made it possible to obtain a metal matrix composite. Its matrix consisted of martensite grains divided into “laths” 0.2–2 μm thick. When high-speed steels are tempered, nanosized carbide compounds of the Me_3_C, Me_7_C_3_, Me_23_C_6_, M_6_C, MeC [24] types or complex carbides, for example (Cr, Fe)_7_C_3_ [25], are precipitated. Apart from the carbide phases, (Fe, Co)_7_W(Mo)_6_ type intermetallics, MeX or Laves phases [26] can be formed. The carbide or intermetallic grains of 20–50 nm in diameter are uniformly distributed in the steel. They prevent recrystallization when the tools are heated, which enables them to be applied in extreme working regimes. Carbon steels retain their mechanical properties only up to 200 °C, while high-speed steels have stable structure up to 600–650 °C. 

**Figure 1:**
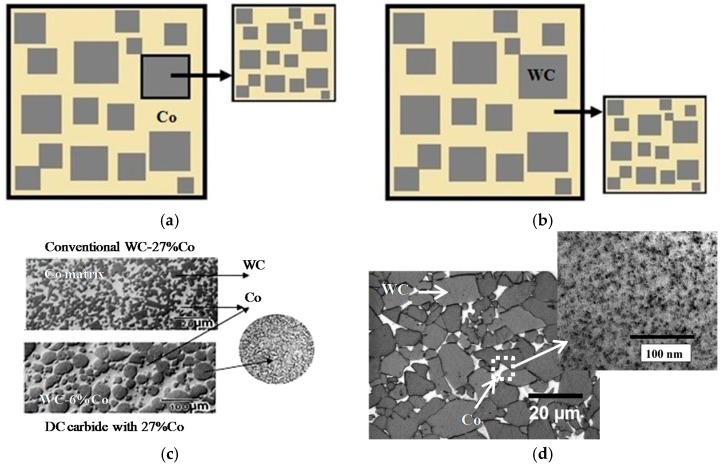
A schematic view (**a**,**b**) and real structures (**c**,**d**) [30,31] of composite cemented carbides: (**a**,**c**) cemented carbide with composite WC-based grains; (**b**,**d**) cemented carbide with composite Co-based matrix.

Cemented carbides have replaced high-speed steels in metalworking and mining. Cemented carbides are metal-ceramic composites that consist of hard tungsten, titanium and tantalum carbide grains located in ductile matrix (binder) based on cobalt or nickel, and have a unique combination of high hardness, wear resistance and toughness [27,28]. By now, a large number of cemented carbide grades have been developed, with diverse combinations of components both in the carbide phase and in the binder. The opportunities to improve cemented carbides by changing their chemical composition have been practically exhausted. That is why formation of nanostructure is a promising approach for enhancing their properties. This approach was implemented in double cemented WC-Co [29,30]. The microstructure of the carbide grains is a scaled-down copy of the structure of the cemented carbide itself, i.e., it consists of carbide grains in a cobalt matrix (Figure 1a,c). In comparison with standard cemented carbides, these materials have higher values of toughness and wear-resistance.

In [31,32], on the contrary, the cobalt binder of cemented carbide was modified. It was reinforced by means of precipitated secondary θ-phase (Co_2_W_4_C) grains 2–5 nm in size (Figure 1b,d). Such material has enhanced hardness, strength and toughness in comparison with its analogs that have the same size of carbide phase grains.

Besides dispersion-hardened composite binders, particle-reinforced cemented carbides are used. Nanoparticles are located incoherently to the matrix. The possibility of enhancing the mechanical properties of WC–Ni hard alloy by introducing SiC nanowhiskers into the binder was examined [33]. The maximal hardness and bending strength values were achieved at the content of nanowhiskers of about 0.5 wt %. The high mechanical properties of the cemented carbides with nanomodified structural constituents made it possible to enhance the performance of mining and building tools.

**Figure 2:**
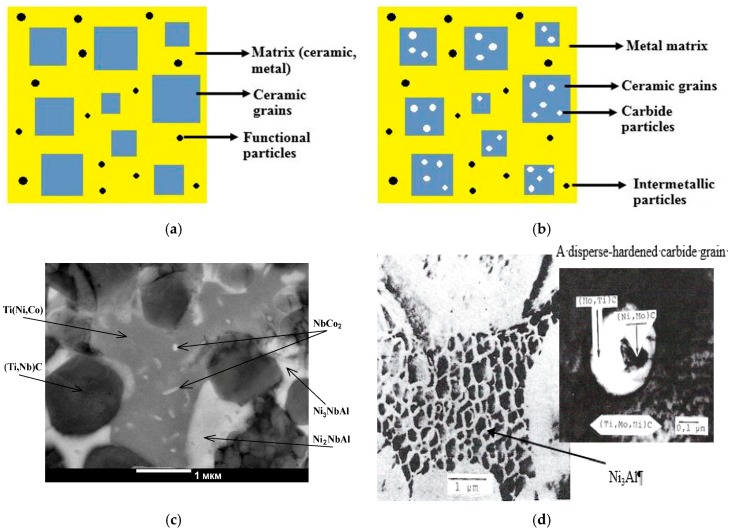
A schematic view (**a**,**b**) and real microstructures (**c**,**d**) [34,37] of ceramic materials: (**a**,**c**) with disperse-hardening binder; (**b**,**d**) with simultaneously reinforcing the grains and the binder.

Ceramic materials for metalworking are widely used tool materials based on aluminum, silicon and titanium oxides, carbides or nitrides. The development of ceramics with disperse-hardened binders is one of the promising ways to enhance them; for example, self-propagating high-temperature synthesis (SHS) composite materials based on Ti-Zr-C and Ti-Nb-C with Ni–Al–Co–Cr binder, where intermetallic phase grains 50–70 μm in size (Figure 2a,c) are precipitated during annealing. Precipitation of the intermetallic phase increases the material hardness, wear resistance and oxidation resistance [34]. Also, alloys with a hierarchical structure not only in the metallic binder, but also in the carbide grains: the so-called “STIMs” (synthetic hard tool materials), were obtained using the SHS method [35,36].

STIM-5 grade alloy is a new class of ceramic materials with a hierarchical structure and the effect of simultaneous disperse reinforcement of the carbonitride grains by precipitating an excess molybdenum carbide phase from oversaturated (TiMo)CN solid solution, as well as the reinforcement of metallic Ni-Al-Mo-Nb-Co-Cr binder as a result of precipitating the Ni_3_Al γ’-phase (Figure 2b,d). Cutting plates made from STIM-5 alloy have a superior cutting performance in finishing and semi-finishing regimes of steels machining, compared to industrial cemented carbides based on WC [37,38].

Nanocomposites are also successfully applied for cutting tools based on SHMs. Over the last decade, the advantage of diamond-cutting tools with a composite binder—a metal matrix based on copper, iron, cobalt, nickel and their alloys—has been demonstrated [39,40,41,42]. The matrix was reinforced with nanoparticles of refractory WC, ZrO_2_, Al_2_O_3_, Si_3_N_4_ compounds, nanodiamonds, etc. The introduction of nanoparticles ensures reinforcement of the composite material according to the Orowan mechanism (impeding the movement of dislocations). In this case, bending strength rose by 20–50% and hardness increased by 15–20%. Enhancement of the binder mechanical properties strongly influences tool productivity and service life. Firstly, the particle-reinforced binder wears less intensely, leading to an increase in the SHM grain efficiency ratio [43]. Secondly, as the bending strength rises, the binder’s capacity to retain the SHM grains increases. The coefficient of correlation between these characteristics amounts to 0.979 [44]. Thirdly, the nanoparticles present in the binder are capable of performing a protective function—the prevention of chemical and mechanical wear of SHM grains. It was noted that WC nanoparticles slow down the diamond graphitization process [45], which was related to the fact that they block the contact area between diamond and binder. Moreover, carbon atoms diffuse across the grain boundaries into the binder volume faster and do not form a low-strength layer on the diamond surface. This effect is essential, and positively affects diamond stability. It was concluded that the diamond crystals can be spontaneously coated by tungsten carbide directly during sintering of the diamond-containing materials that comprise WC nanoparticles [46,47]. Oxygen impurity presenting in the plasma-chemical WC nanopowder plays an important role in this process. It was established, that the tungsten carbide coating forms via a gas-phase transport mechanism and chemosorption of volatile WO_3_ tungsten oxide on local areas of graphitized diamond surface, with a subsequent reduction and carbide formation. The obtained coating leads to enhancement of diamond-retention and tool life. 

Nanostructured wear-resistant coatings are a widely used way to enhance the performance of cutting tools. Multilayer and multicomponent coatings have the highest mechanical properties, wear-resistance and adhesion to the substrate. Wear-resistant coatings can be divided into the following classes: hard materials (borides, carbides, and nitrides of transition metals); covalent hard materials (Al, Si borides, carbides and nitrides as well as diamond coatings); and ceramic hard materials (Al, Zr, and Ti oxides, etc.) [48]. Depending on the operating conditions, the coatings may possess a different architecture with alternating layers of hard refractory compounds, metals and solid lubricants. Such coatings are divided into the following groups: hard/hard (combinations of carbides, borides, nitrides, etc., for example, TiC/TiB_2_, TiN/TiB_2_); hard/soft (carbide/metal, for example, B_4_C/W, SiC/Al); soft/soft (metal/metal, for example, Ni/Cu and solid lubricant/metal, for example, MoS_2_/Mo, WS_2_/W) [49]. The total thickness of the multilayer coatings may reach 2–5 μm, while the thickness of an individual layer usually amounts to several nm. The deposition of coatings on a tool-working layer increases its productivity up to 200% during cutting, its service life up to 10 times when cutting steels, and corrosion resistance, etc. [50,51,52,53].

Composite materials based on bulk metal glasses (BMG) modified with nanosized inclusions of metallic or ceramic phases are one of the most promising classes of modern materials that can be used in machining. By now, a large number of different BMGs based on Zr, Ti, Mg, Al, Cu, Ni, Pd have been developed [54,55,56,57,58,59,60]. At first, these materials were produced through ex situ processes, in which solid nanoparticles were added to molten metals or alloys, followed by quenching [61,62], or by powder metallurgy techniques (mechanical alloying of powdered components) [63]. The main requirement for nanoparticles was the absence of solubility in the matrix. For example, BMGs with a Mg matrix were reinforced by MgO, CeO_2_, Y_2_O_3_ nanoparticles, and those with a Zr matrix reinforced by CaO, ZrO_2_, ZrC, W, and Ta nanoparticles, etc. Later, BMGs were produced through in situ methods, which involved the precipitation of crystalline phases during cooling of solid solutions [64,65].

BMGs are well-known for their unique combination of high mechanical properties: strength, hardness, and Young’s modulus [66]. The features of their deformation and failure behavior are associated with the formation of highly localized shear bands under loading. Shear bands located in plastically soft areas are suppressed in areas with higher stiffness [63]. The localization of shear bands is the result of a rapid dilation accompanied by intense shear deformation of short-range ordered clusters. The spreading of localized shearing events occurs around shear transformation zones, and leads to formation and accumulation of a free volume. Deformation in the shear bands results in intense plastic flow. Thus, a small amount of them is enough for dramatic failure of the material.

The presence of nanosized crystalline inclusions within BMGs makes it possible to significantly enhance their mechanical properties and to improve tensile ductility and fracture toughness by several times [67,68]. The arrangement of crystalline nanoparticles can change the deformation mechanism of BMGs. Up to a certain concentration of nanoparticles, they remain isolated and uniformly distributed in the matrix. Since their size is much smaller than the distance between shear bands, they enhance resistance to the plastic flow and increase viscosity within the shear bands. As a result, the propagation of shear bands is retarded, which leads to an increase in the plasticity of the material [69,70].

The high mechanical properties of BMGs indicate high potential for using them in machining different materials. However, at present, their application is limited due to the complication of producing bulk samples and possible devitrification processes (crystallization at heating). Thus, utilization of BMGs in machining is possible in the case of low temperature generation (less than 500 K) in the contact zone of the tool and workpiece [71]. However, it is known that they are used in blades [71] and other types of cutting tools [72,73] and their further application may be associated with metal-cutting.

The unique properties of metallic glasses can be utilized in materials with amorphous metallic coatings, including those hardened with nanosized crystalline inclusions [74,75,76].

Thin-film metallic glasses (TFMGs) can be formed by several methods of physical vapor deposition (PVD), primarily magnetron sputtering [77]. In order to control the film composition, the sputtering target is usually designed to contain a complex form with slices made of different elements, or co-sputtering by two or three sources can be used. For nanocrystals to be formed in the structure of thin film, the TFMG samples are heated to temperatures higher than the crystallization temperature [78].

Alongside their special magnetic and electric properties, features of TFMGs containing crystalline inclusions are their high mechanical and fatigue properties, and also their advantage over metallic glasses coatings with completely amorphous structures [79,80,81]. It has been shown that the hardness of coatings in a Zr–Cu–Al–Ni system with the same composition after annealing at the crystallization temperature and higher increased by 50% [78]. The increase in hardness was attributed to the combined effects of the composite structure and free-volume annihilation due to structure relaxation [79].

In addition, TFMGs have a very smooth surface with low roughness, which impedes the generation of cracks when the sample is deformed.

## 3. Carbon Nanotube-Doped Nanocomposites for Machining

Carbon nanotubes (CNTs), with their high mechanical properties, are of a special interest for use as metal matrices fillers [82]. CNTs have an average elastic modulus of 1000–2000 GPa, the average bending strength of multiwall CNTs (MWCNTs) amounts to 6–22 GPa, and their tensile strength reaches 11–63 GPa [83]. MWCNTs can be successfully applied in order to reinforce cemented carbides. The introduction of less than 0.5% of MWCNTs into nano-WC-7%Co cemented carbide enhances the material’s mechanical properties and “hardness-to-toughness” ratio [84]. Preliminarily WC coating deposition on the carbon nanotubes makes it possible to obtain a more homogenous structure, increase the adhesion between the matrix material and nanotubes, and decrease the porosity of the nano-WC-10%Co cermet [85]. Besides, tungsten carbide, silicon carbide, which prevents oxidation of the nanotubes, may also be used as a coating [86,87].

Introduction of up to 5% MWCNTs coated by SiC into a ceramic silicon carbide matrix considerably increases material hardness and toughness. Besides, this composite material has elastic behavior due to the bridging effect of the MWCNTs [88]. 

Carbon nanotubes are also used to increase the performance of diamond tools. If CNTs are introduced into a Ni coating during the manufacture of electroplated diamond tools, the hardness and yield strength of the nickel matrix increase substantially. As a result of calculations, it has been shown that enhancement of the mechanical properties leads to an increase in retention of the diamonds by a factor of 1.3. After testing, the surface of the tool-working layer with nickel binder was severely worn as compared to the tool with Ni-CNT binder, which is evidence that the binder wear-resistance and diamond retention was enhanced. The calculations were confirmed by a substantial (up to 8 times) growth of the tool life in hole-drilling of fused silica and side machining of white plate glass [89].

**Figure 3:**
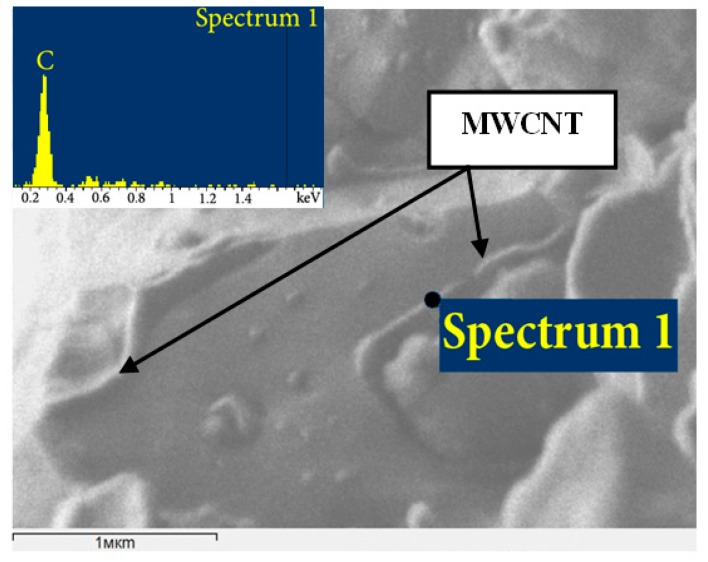
Carbon nanotubes in the hot-pressed binder reinforced with multiwall carbon nanotubes (MWCNTs) [92].

The results of the MWCNTs effect on the mechanical properties and performance of an iron-based binder for diamond-cutting tools were investigated in [90,91,92]. It was established that the increase in the hardness, strength, and Young’s modulus observed at an optimal concentration of carbon nanotubes (less than 0.1%) leads to a decrease in grain size. The grain-size decrease is related to the binder recrystallization process being impeded due to presence of the nanotubes at grain boundaries (Figure 3).

According to the results of comparative diamond core drill tests of steel-reinforced concrete with granite filler, it was established that tool productivity increased by 50%–70% compared to the non-modified binder (Table 1). 

Thus, the application of carbon nanotubes for the modification of metal matrices for cutting tools is a promising way to further enhance the performance of the diamond tools. The enhancement of mechanical properties is related to the decrease of matrix material grains along with the slowing down of the process of coalescence of the metallic grains.

Moreover, use of single-layer carbon nanotubes as the reinforcing phase—due to their higher mechanical properties compared with multilayer ones—and preliminarily coating of the nanotubes in order to prevent their oxidation and interaction with the matrix material, can be implemented for the development of new nanocomposite materials with enhanced properties. 

## 4. hBN-Doped Nanocomposites for Machining

Hexagonal boron nitride (hBN) is widely used as a solid lubricant that operates at elevated temperatures [93] and is added to cooling or lubricating liquid. In this case, hBN nanoparticles form an antifrictional layer on the surface of the treated item, which significantly decreases the friction coefficient [94,95].

T. Ohji et al. showed for the first time the effectiveness of hBN-nanoparticle modification of aluminum oxide or silicon carbide materials doped with a little B_2_O_3_ or SiB_6_ [96]. Such materials were obtained by reactive hot pressing in a nitrogen environment. Interaction of B_2_O_3_ and SiB_6_ with nitrogen at high temperatures led to the formation of hBN nanoplatelets with a diameter of 200 nm and 60–80 nm thick. The reinforcement of the ceramics was achieved by the formation of an ultradisperse structure.

**Figure 4:**
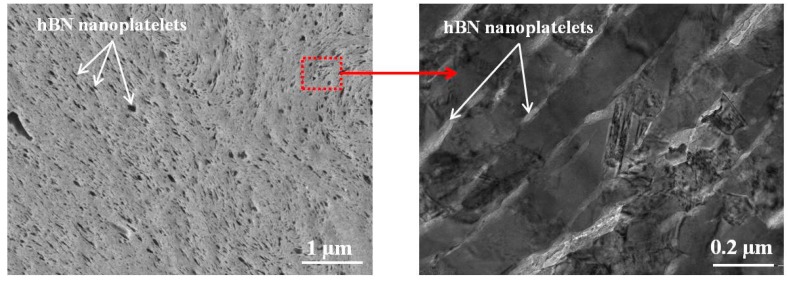
Microstructure of the composite particle after planetary ball milling recorded by transmission electron microscopy.

A similar approach to improvement of tool materials by alloying them with hBN particles was successfully applied in [51,95,96]. It was shown that reinforced metal matrix composites can be obtained by mixing the powders in a planetary ball mill and subsequent hot pressing. During the interaction with the milling agents, the micron-sized hBN powder separates into nanoparticles 70 nm in size and 15–18 nm thick, which are uniformly distributed within the volume of the material [97] (Figure 4). 

**Figure 5:**
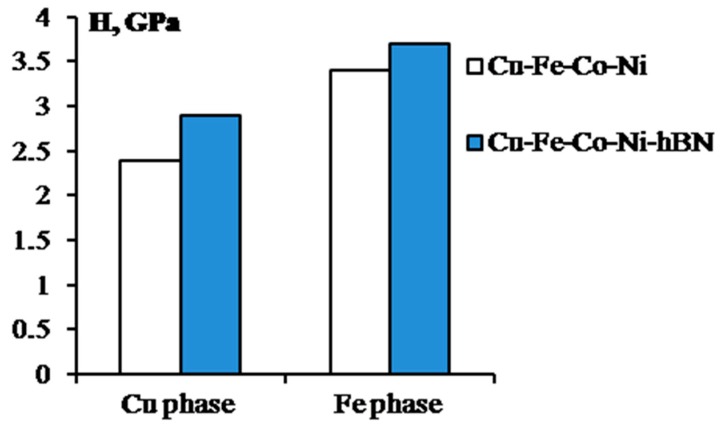
Hardness of Cu- and Fe-based phases in hot-pressed samples of Cu–Fe–Co–Ni and Cu–Fe–Co–Ni-hBN binders.

The mechanical properties of the Cu–Fe–Co–Ni binder used for the diamond tools are enhanced by 15–20% when modified with hBN nanoparticles. A decrease of the average size of metal matrix grains is the main mechanism of this reinforcement. Hexagonal boron nitride is chemically inert relative to the binder materials used during the production of diamond tools. hBN nanoparticles located at the boundaries of the matrix grains prevent diffusive processes and recrystallization during the sintering or hot pressing. On average, the grains of the binder modified with hBN nanoparticles are 1.5 times smaller than those of the initial binder [98]. The introduction of nanoparticles affected the mechanical properties of the whole composite material and the phases comprising it. The effect of hBN on the hardness (H, GPa) and elastic modulus (E, GPa) of the phases based on copper and iron was studied for the Cu–Fe–Co–Ni binder [99]. The hardness was found to be higher by 10–20% in the case of the nanomodified alloy (Figure 5). The enhancement of the properties was explained by the Hall–Petch effect.

Due to high mechanical properties of the binders modified with hBN nanoparticles, an enhanced performance of the diamond tools was observed. For example, diamond-cutting wheels with nanomodified Cu–Fe–Co–Ni binder had a productivity in cutting cast iron that was higher by 80% (Table 2) [98]. Authors [100] have related enhancement of the tools’ productivity to the increase in the diamond retention strength and the preservation of the cutting capability of the diamond grains.

Besides the positive influence on the diamond retention strength, hBN nanoparticles enhance the compactibility of the powder mixtures during cold and hot pressing [97], decrease the binder wear, and prevent seizure at the cutting tool-workpiece interface at high temperatures. hBN nanoparticles cover the surface of the diamond grains, thus decreasing the area of contact with metal catalysts (iron, cobalt, nickel) and prevent the diamond from graphitization during hot pressing [101].

Thus, hBN is a promising material for the development of nanocomposites used for machining tools. Introduction of hBN nanoparticles leads to a significant increase in the strength of tool materials, despite the low mechanical properties of hBN, which is not typical for the reinforcing phase. Due to the possibility of obtaining nanocomposites with uniform distribution of the nanomodifier in various ways (reactive hot pressing or ball milling combined with conventional hot pressing), hBN can be considered as a high-tech additive.

## 5. A Micromechanical Model of the Reinforced Metallic Matrix

### 5.1. Metallic Fe–Cu Binder Reinforced with CNT Particles

**Figure 6:**
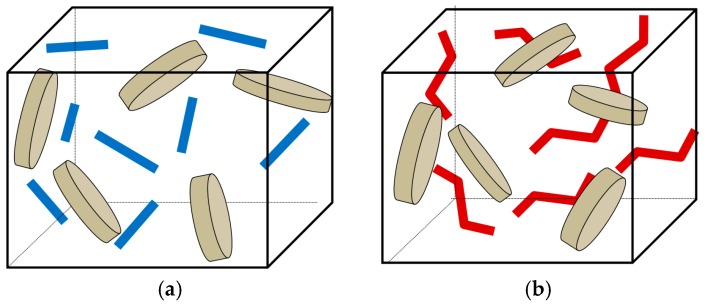
A schema of unit cell model Fe/Cu bonds: (**a**) with straight MWCNTs; (**b**) with zigzagged MWCNTs.

For the development and optimization of nanoengineered metallic materials, microstructural computational models can be used. In order to analyze the role and effect of the MWCNT reinforcement on the mechanical and damage behavior of the metal matrix of the tool, a series of micromechanical computational models has been developed. The material was considered as three-phase material, with elongated disc-shaped iron inclusions, a high aspect-ratio carbon nanotube, represented by cylinders, and a cupper matrix. The volume content of iron- and copper-based phases in the MMC was considered to be roughly constant (87% Fe, 13% Cu). The MWCNTs were randomly located and also randomly oriented. Two cases were considered: ideal cylindrical MWCNTs (represented in the 2d version as randomly oriented rectangles) and zigzagged MWCNTs with 4 sections each (each section is 90 degrees inclined to other sections). Schema of unit cells with straight and zigzagged nanoparticles are shown in Figure 6 [53,92,98]. The computational models were generated using the Python command language, and run on the commercial finite element code Abaqus. 

### 5.2. Effect of MWCNT Reinforcement on the Mechanical Properties of the Binder and Tool Wear

**Figure 7:**
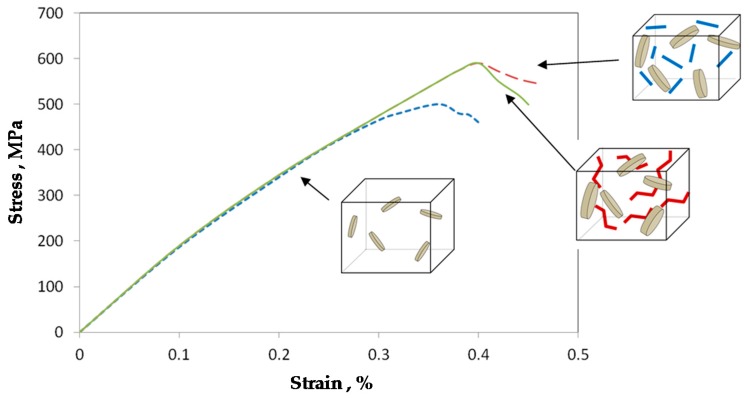
The stress–strain curves.

A series of computational studies of the deformation behavior of MMC binders were carried out. The unit cells were subject to displacement tensile load on the upper border. The experimentally obtained stress–strain curves were introduced in the model. For the estimation of damage initiation, the ductile damage criterion was assumed for both Cu- and Fe-based phases. As expected the highest stress concentration is on the tips of long Fe-based grains in the MWCNT-free binder [92]. In the MWCNT-reinforced materials, the highest stresses are in MWCNTs and around them. The stress level in the straight MWCNTs is ~40% higher in the material with straight tubes than with zigzagged tubes. The straight MWCNTs shield and concentrate stresses near their ends, while zigzagged MWCNTs cause more complex stress distribution, with local stress concentrations near corners of “zigzags”. Figure 7 shows the obtained stress–strain curves. As noted in [98], MWCNT reinforcement influences the mechanical behavior of the metal composites via the reduction of grain sizes, not via the reinforcement or stress-localization mechanisms. 

Let us consider how such binder modification can influence the performances of the grinding tools. Assuming that the stronger binder allows higher size of the grains above the binder, and estimating the material removal rate in grinding as Q = abv, where a is depth of a cut, bis width of contact, and vis cutting rate. Let us evaluate the balance of forces, removing a diamond grain from the binder. The chip thickness/depth of unit grain cut can be calculated as: t = d − Δ − l, where d is grain size, l is depth of grain embedment in the binder, and Δ is the distance between the binder and work material surface. Assuming the force of the grain P_g_ is proportional to the depth of unit grain cut t (P_g_~τ_wm_td, where τ_wm_ is shear strength of work material), and the force P_b_ keeping the grain in the binder is proportional to the binder strength σ and the depth of grain embedment in the binder l: P_b_~σld = σd (d − Δ − t), and solving the force balance equation, we can see that the chip thickness is an increasing function of the strength of the binder: t ~ σ (d − Δ)/( σ + τ_wm_). As can be seen from this analysis, the material removal rate is an increasing function of the binder strength. Thus, it can be expected that the increase of the binder strength caused by the MWCNT reinforcement should lead to the comparable increase in the material removal rate and drilling efficiency. 

### 5.3. 3D-Modelling of Real CNT Shapes in Matrix

**Figure 8:**
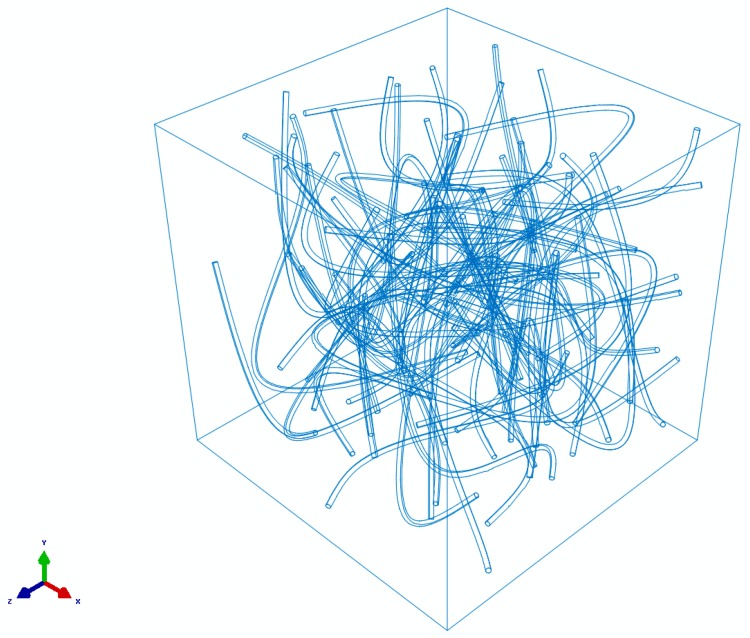
A 3D unit cell with snake-like multisegment CNTs.

In order to analyze the effect of real shapes of the CNT particles versus idealized cylindrical shapes, 3D unit cell models were generated and tested. To implement realistic shape features in the computational models, instead of idealized “cylindrical” nanoparticles, the CNTs were modelled using a sweeping approach [102]. The coordinates of the initial point of each NC fibril was given by three random numbers inside the cell determined using a random number generator [103,104,105]. Then the next point was defined, by defining (again, randomly) two angles. The location of each following turning point was defined by newly generated random angles (now, in a given range). After the points were generated, wires were plotted through the points, and round sections of a given diameter were swept through each array of the points. The random sequential absorption/RSA algorithm was applied sequentially to each new CNT segment. Figure 8 shows an example of such a unit cell.

The effect of real, complex shapes of carbon nanotubes/CNTs on their reinforcing function and damage behavior in nanocomposites was studied using test material [102]. Comparing the realistic (snake-like) and idealized (cylinder-like) models of the nanocomposites, different load distributions were observed. In the case of the straight CNT cylindrical models, only CNTs oriented in the load direction give rise to stress concentrations, and these stress concentrations manifest themselves at the CNT ends. In the case of the snake-shaped CNTs, only the sections of a CNT that are oriented in the load direction show high stress concentrations, which means that only a part of the CNTs is actually carrying the load. 

### 5.4. Metallic Binder Reinforced with hBN Particles

To estimate the influence of the binder structure on performance, a micromechanical analysis of the mechanical properties of the binder with and without nanoreinforcement was carried out. hBN platelets were taken as discs sized at 18 nm (thickness) × 72 nm (radius). The Young’s modulus of the hBN platelets was taken as 675 GPa (see www.panadyne.com). To evaluate the effect of the nanoreinforcement on the mechanical properties, the micromechanical model of Halpin–Tsai [106] modified by Lewis and Nielsen [107] was used. The elastic properties of the nanoreinforced FeCo phase are calculated using the Halpin–Tsai equation for aligned platelets:

(1) $$E=E_{FeCo}\left(\frac{1+\zeta \eta v_{hBN}}{1-\eta v_{hBN}}\right);\eta =(E_{hBN}/E_{FeCoi}-1)/(E_{hBN}/E_{FeCoi}+\zeta )$$

where *E* is the Young’s modulus, ζ and η are the fitting coefficients, and v_CuNi_ is the volume content of the hBN phase, 70%. The estimated value of the Young’s modulus for the Fe–Co matrix with the hBN-reinforced material is 181.4 GPa. This value is sufficiently lower than the value of the Young’s modulus for the Fe–Co-hBN matrix (194 MPa). Practically, it means that the higher mechanical properties of the nanomodified binder are controlled not only by the mechanical reinforcement effects but also by the decreased grain size of the material when manufactured with nanoadditives.

**Figure 9:**
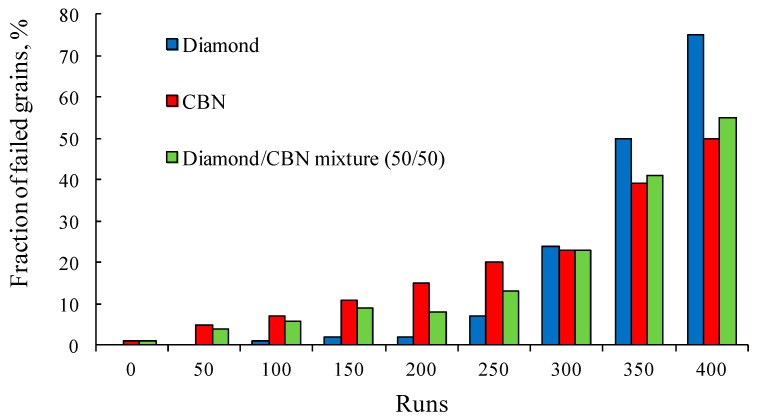
Results of the grain failure simulations: diamond, cubic boron nitride (CBN), and total grain failure (diamond pulled out, CBN cracked).

Figure 9 shows the fraction of the torn diamond grains and of the total grains in the binders. One can see that the difference is quite visible, especially after a high number of runs.

Thus, the micromechanical modelling of the metal matrix materials makes it possible to assess the tools’ performance without significant time and financial expenses by determining the mechanisms of their deformational behavior and failure of the nanocomposite binders. 

## 6. Wear-Resistance of CNT- and hBN-Containing Nanocomposites

Metallic Cu–Fe–Co–Ni, Fe–Co–Ni, and Fe–Ni–Mo binders were used to investigate the influence of carbon nanotubes and hBN nanoparticles on wear-resistance of tool materials. The tribological experiments were carried out using the automated Tribometer friction machine manufactured by CSM Instruments (Switzerland) with a pin-on-disk scheme, under the following conditions: track radius of 6.8 mm, applied load of 2 N, distance of 214 m (5000 cycles). A 3-mm alumina ball was used as a counterpart. 

**Figure 10:**
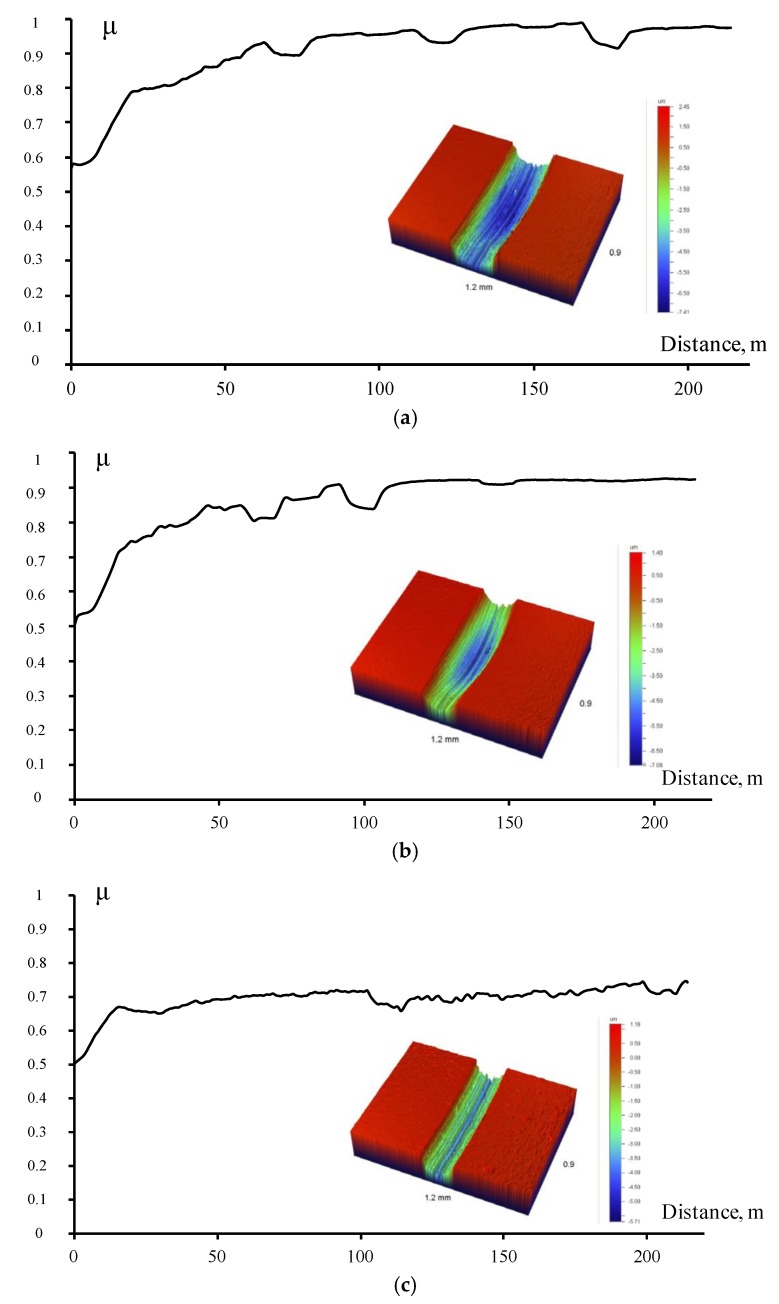
Results of tribological tests: (**a**) Fe–Ni–Mo; (**b**) Fe–Ni–Mo-CNT; (**c**) Fe–Ni–Mo-hBN samples.

The dependence of the friction coefficient vs. distance and corresponding wear tracks are shown in Figure 10. It was found that specific wear of the binder modified with CNTs and hBN nanoparticles was lower by two times than that of the initial binder (8.3–8.6 and 15.6 × 10^−5^ mm^3^/N/m respectively). Introduction of hBN particles into the binder led to a friction coefficient decrease from 0.9 to 0.7.

The results of multiple tribological tests of metal matrix nanocomposites that contain CNTs and hBN demonstrate that application of these modifiers makes it possible to enhance the wear-resistance of the tools when machining materials with a high hardness.

## 7. Conclusions

Nanocomposites attract wide attention in relation to modern tool materials because of their unique structures, high mechanical properties and wear-resistance. Ceramics, cemented carbide and metallic binders modified with carbon nanotubes and hBN nanoparticles have a special role. The positive effect from CNTs, and hBN modification, includes the enhancement of the material hardness and yield strength, which leads to an increase in the wear-resistance and tool life.

Regardless of the fact that the machining tool industry is developing rapidly, manufacturers are still working on novel materials that are reasonably priced, strong, and can successfully replace today’s best benchmark specimens. All these efforts are aimed at obtaining materials that can provide the highest possible levels of productivity.

In this context, the upcoming development of nanocomposites for machining tools can be associated with the implementation of materials with hybrid structures. The modification of metallic and ceramic matrices by the addition of nanoparticles of two or more types allows the mechanical properties and the performance of materials to be enhanced dramatically. 

Hybrid modification with carbon nanotubes and hexagonal boron nitride, as well as studies of interaction of the dislocation front with the nanoparticles upon the deformation of such hybrid materials, are promising ways to further develop machining tools with enhanced properties. Mathematical modeling will facilitate the creation of hybrid nanomaterials with an optimal combination of their mechanical properties, which will ensure enhanced performance of the tools.

Moreover, significant efforts must be made to eliminate what are understood to be the weak points of modern top-level tool materials (for example, the technological complexity and brittleness of ceramics, CBN, BMGs). Solving this problem can possibly lead to the design of a new class of multi-functional material.

## Figures and Tables

**Table 1:** Results of comparative testing (tool life and drilling speed) of diamond core drills, 2% and 10% (a and b) steel content in the reinforced concrete. Adapted from [92].

Binder Composition	Tool Life, m		V_drill_, cm/min	
	a *	b	a	b
V21	12.0 ± 1.0	3.4 ± 0.3	4.0	1.51
	11–13	3.1–3.7		
V21 + 0.1%MWCNT	13.2 ± 1.0	3.2 ± 0.3	6.4	1.72
	12.2–14.2	2.9–3.5		

* a (2%) and b (10%) steel content in the reinforced concrete.

**Table 2:** Performance of diamond-cutting wheels with Cu–Fe–Co–Ni binders. Adapted from [98].

Binder Composition	Productivity, cm^2^	Cutting Speed, cm^2^/h
Cu-Fe-Co-Ni	950	220
Cu-Fe-Co-Ni-hBN	1600	320
